# Surgical risk factors for technical survival of peritoneal dialysis catheters

**DOI:** 10.1007/s00423-025-03901-7

**Published:** 2025-11-10

**Authors:** Lukas Pollmann, Nicola S. Pollmann, Claudius Jürgens, Fedai Özcan, Alexandra Brinkhoff, Maximilian Schmeding

**Affiliations:** 1 Department of General and Visceral Surgery, Klinikum Dortmund gGmbH, University Hospital of the University Witten/Herdecke, Beurhausstraße 40, 44137 Dortmund, Germany; 2 Department of General-, Visceral and Transplant Surgery, University Hospital Jena, Am Klinikum 1, 07747 Jena, Germany; 3 Department of Nephrology, Witten/Herdecke University, Klinikum Dortmund gGmbH, Beurhausstraße 40, 44137 Dortmund, Germany

**Keywords:** Peritoneal dialysis, Peritoneal dialysis catheter, PD catheter removal, Technical survival, Postoperative revision, End-stage renal disease

## Abstract

**Purpose:**

Peritoneal dialysis has been demonstrated to be a cost-effective modality of dialysis treatment, providing a greater quality of life in comparison to hemodialysis. However, complications associated with the peritoneal dialysis catheter (PD catheter) can lead to increased patient morbidity and thus the necessity of PD catheter removal. While prior studies have identified patient-related risk factors, the impact of various surgical risk factors on technical survival is yet to be elucidated.

**Methods:**

A retrospective, monocentric cohort study was conducted including all patients who underwent PD catheter implantation through an open surgical technique utilizing a small surgical incision above the rectus abdominis muscle from January 2010 to March 2022. The technical survival of PD catheters was observed retrospectively over a period of three years and the reasons for PD catheter removal were summarized. Furthermore, Cox regression analysis was conducted to evaluate potential risk factors for a reduced technical survival.

**Results:**

A total of 340 patients were included, and a median PD catheter functionality of 980 days was presented in this study. The main reasons for PD catheter removal included infectious complications and mechanical malfunctions. Postoperative revision was identified as a significant risk factor for a reduced technical survival.

**Conclusion:**

PD catheter implantation through a small surgical incision showed a high long-term functionality regardless of prior abdominal surgery, prior PD catheter implantation, or the necessity of adhesiolysis. Only postoperative revision was identified as a significant risk factor for PD catheter removal.

**Trial registration:**

The study was registered in the German clinical trial database (Application number DRKS00036575, registration date 19.05.2025).

**Graphical abstract:**

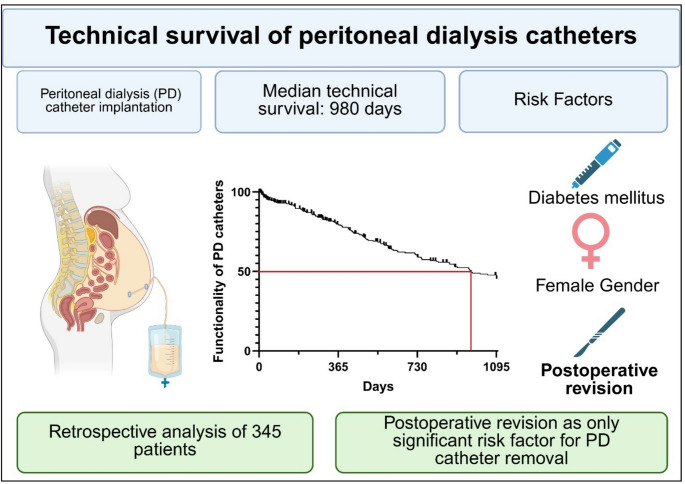

**Supplementary Information:**

The online version contains supplementary material available at 10.1007/s00423-025-03901-7.

## Introduction

The modality of renal replacement therapy is of major importance for patients with end-stage renal disease. In the case of long-term treatment, the necessity of frequent hemodialysis in a specialized facility or peritoneal dialysis utilizing a peritoneal dialysis catheter (PD catheter) needs to be discussed [1, 2]. Compared with hemodialysis, PD as home dialysis may offer an improvement in quality of life as it can be performed autonomously by the patient. Furthermore, PD was found to be a more cost-effective solution compared with hemodialysis [3]. Nevertheless, various infectious and non-infectious complications associated with the utilization of catheters have been identified necessitating PD catheter removal. Consequently, a decline in the functionality of PD catheters was observed, along with a decrease in the technical survival rate [4–6]. The two-years functionality rate varied from 60% [7] to 91% [6] in previous studies. In particular, catheter-associated peritonitis [4, 8] increases morbidity and mortality, which frequently leads to the removal of the PD catheter. Especially, diabetes mellitus has been shown to increase the risk of PD catheter infection and peritonitis, thus lowering the rate of PD catheter functionality [5, 9]. Furthermore, older age [7] and female gender [10] have been identified as patient-related risk factors associated with diminished PD catheter functionality.

In addition, postoperative hematoma, obstruction, tip migration or catheter leak as non-infectious complications led to the removal of the PD catheter [6]. While laparoscopic implantation showed a lower migration rate and less postoperative adhesions compared to open implantation, the rate of postoperative hematoma was higher compared to open implantation [11–14].

Furthermore, several technical factors have been previously analyzed, including catheter type [15] and the impact of previous abdominal surgery and intraperitoneal adhesions on PD catheter functionality [16, 17]. These previous studies suggest that peritoneal dialysis is possible after abdominal surgery with intraperitoneal adhesions without compromising catheter functionality or increasing the rate of surgical revisions. Furthermore, a higher BMI was not associated with reduced PD catheter functionality in previous research [18], although a higher rate of PD catheter associated peritonitis has been discussed [19]. In summary, previous studies have identified several risk factors for reduced PD catheter functionality. Nevertheless, the influence of various surgical risk factors on catheter survival, including previous PD catheter implantation and the rate of surgical revisions due to postoperative malfunction, remains to be elucidated.

## Materials and methods

### Data acquisition and ethical statement

A retrospective, monocentric cohort study was conducted including patients with end-stage renal disease who underwent PD catheter implantation from January 2010 to March 2022. The functionality of the PD catheter and patient survival were recorded retrospectively over a period of three years. Patients under the age of 18 were excluded from the analysis. Subsequently, the patient-related risk factors of age, diabetes mellitus, gender, BMI and previous abdominal surgery, were determined. In addition, previous PD catheter implantation and revision due to dysfunction during hospitalization were recorded. Finally, the need for intraoperative adhesiolysis was determined.

The study was approved by the institutional ethics committee (Ethics Committee Witten/Herdecke; application number: S-66/2025, date of approval: 11.03.2025). In addition, the study was registered in the German database for clinical studies (application number: DRKS00036575).

### Operative procedure

For PD catheter implantation, an Oreopulus-Zellermann-PD catheter [20] with a straight intraperitoneal segment was utilized. Initially, a small incision of approximately eight centimeters was made over the left or right rectus abdominis muscle below the umbilicus. The abdominal wall fascia was then incised and the rectus abdominis muscle bluntly divided. Subsequently, the peritoneum was opened, and the PD catheter was introduced. The peritoneum was sutured watertight around the PD catheter. Finally, the PD catheter was passed transrectally through the abdominal wall and the abdominal wall fascia and skin were closed. In the presence of intraperitoneal adhesions hindering implantation, laparoscopic adhesiolysis was performed as a hybrid procedure prior to insertion of the PD catheter.

### Statistical analysis

First, the patient characteristics and potential risk factors mentioned above were summarized in the retrospective cohort. Subsequently, PD catheter functionality was depicted over the 3-year follow-up period using a Kaplan-Meier curve with censoring for death and loss-of-follow-up. Patients who received a kidney transplant, or those who experienced an improvement in renal function resulting in the subsequent removal of their PD catheter, were excluded from the analysis. Finally, a Cox regression was calculated to determine the patient-related risk factors and the surgical risk factors for reduced PD catheter functionality.

**Fig. 1:**
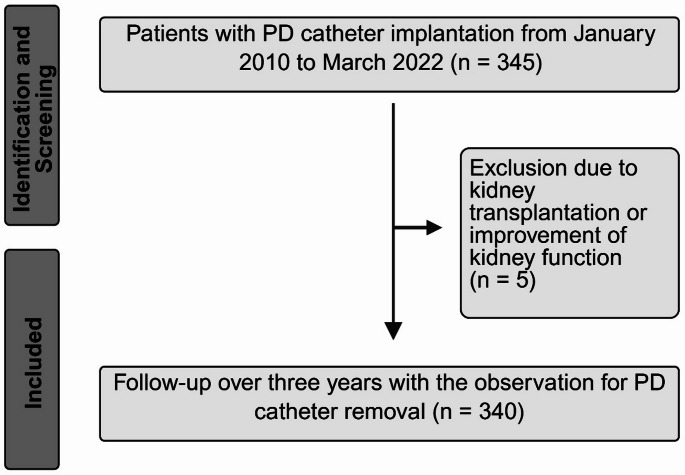
Flow diagram for patient identification, screening and inclusion in the current study

In addition, the reasons for PD catheter removal were summarized. All statistical analyses were performed using Medcalc software (Medcalc Software Ltd); data were visualized using Graphpad software (GraphPad Software, Inc). Biorender (Biorender, Toronto, Canada) was used to create Fig. 1.

## Results

A total of 365 PD catheter implantations were performed in 345 patients from January 2010 to March 2022. The number of implanted PD catheters ranged from 20 implantations in 2019 to 40 PD catheter implantations in 2021 (see Table S1 of the supplementary material). During the observation period, 20 patients underwent a second PD catheter implantation. Five patients were excluded from the analysis. These patients either experienced an improvement in renal function and subsequently had their PD catheters removed, or had their catheters removed after kidney transplantation. The final analysis included 360 catheter implantations in 340 patients, as shown in Fig. 1. The median follow-up in this retrospective analysis was 864 days (95% CI: 489 to 1096 days). During the observation period, 59 patients died without prior removal of the PD catheter.

## Patient characteristics

In our retrospective cohort, the mean age was 61.8 ± 16.2 years (mean ± standard deviation). Most patients were male (57%) and less than half of the patient cohort was diagnosed with a diabetes mellitus type II (42%). Furthermore, the overall BMI of the cohort was 26.9 ± 5.1 kg/m². A total of 85 patients (25% of the entire cohort) had a documented history of abdominal surgery, while 40 patients (12%) had previously undergone PD catheter implantation. However, adhesiolysis was only necessary in 15 patients (4%). The patient characteristics are summarized in Table 1.

**Table 1:** Patient characteristics

	*n* = 340
Gender (% female)	147 (43)
Age (mean ± SD) in years	61.8 ± 16.2
BMI (mean ± SD) in kg/m²	26.9 ± 5.1
Diabetes mellitus type II (%)	142 (42)
Previous operation (%)	85 (25)
Previous PD catheter (%)	40 (12)
Revision (%)	28 (8)
Adhesiolysis (%)	15 (4)

SD = standard deviation

BMI = Body mass index

PD = peritoneal dialysis

### PD catheter removal and possible risk factors

**Fig. 2:**
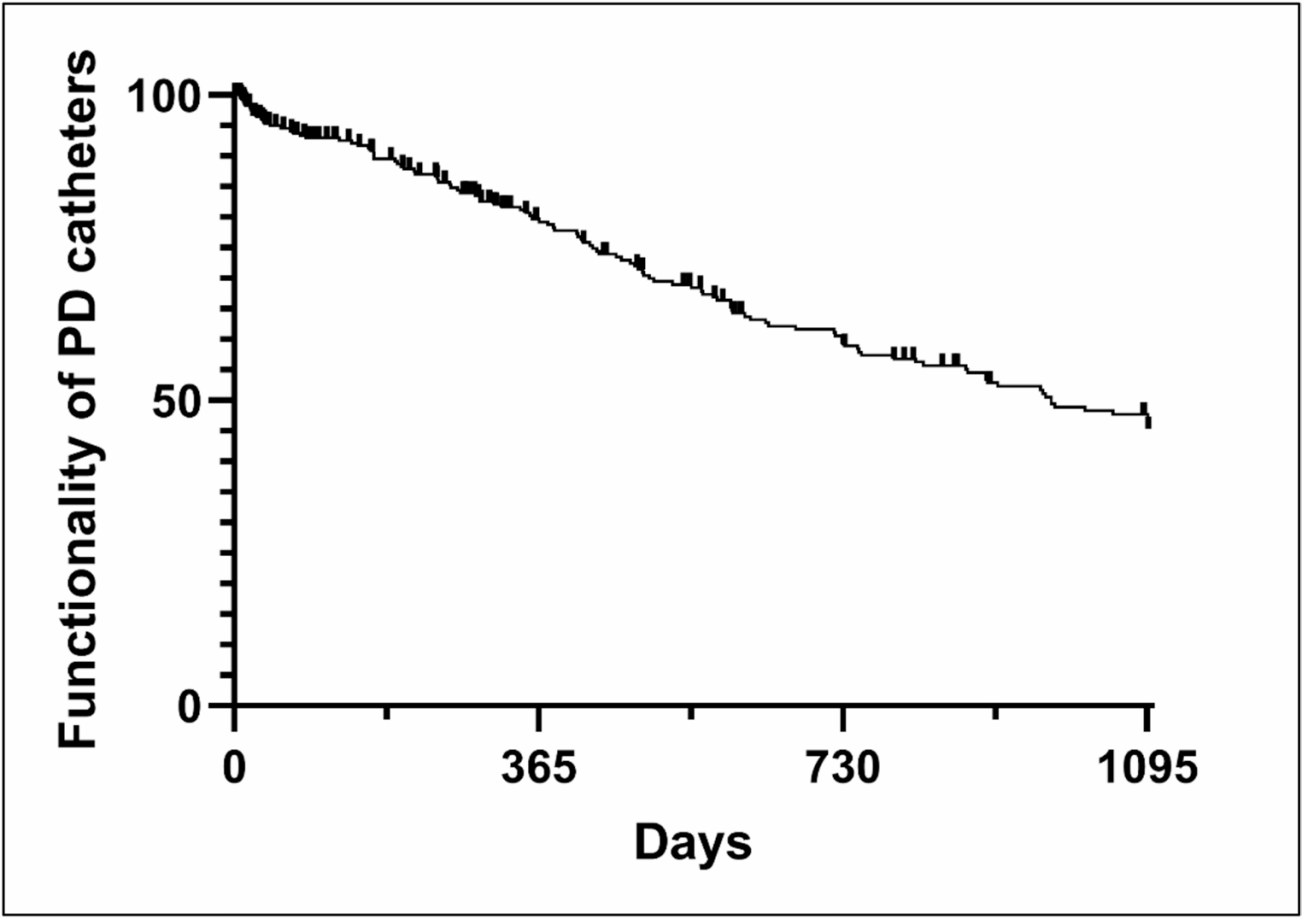
Kaplan-Meier curve illustrating the functionality of PD catheters over time after implantation. The ticks represent loss-of-follow-up

The PD catheter had to be removed in 104 patients during the three-year follow-up period. A total of ten patients underwent a second implantation and subsequent removal of a PD catheter. This resulted in a total of 114 PD catheters being removed during the observation period. Overall, a median PD catheter functionality of 980 days (95% CI: 791 to 1095 days) was observed (see Fig. 2).

Cox regression analysis revealed a significantly lower PD catheter functionality in patients with postoperative revision (*p* = 0.007, hazard ratio of 2.32 [95% CI 1.29–4.28]). In 28 patients, or 8% of the total cohort, revision surgery was necessary due to malfunction of the PD catheter (see Table 1). All revisions were performed laparoscopically. Tip migration was found in seven patients, necessitating laparoscopic revision. Five of these patients underwent laparoscopic catheter fixation. Furthermore, malfunction due to postoperative adhesions was observed in 18 patients. Most of the adhesions were found between the greater omentum and the PD catheter. This required laparoscopic revision in nine patients. Of these patients, four underwent partial omentectomy and two underwent omentopexy. Laparoscopic adhesiolysis alone was performed on three patients. Additionally, five patients had adhesions of the small bowel to the parietal peritoneum involving the PD catheter, and one PD catheter was trapped in inter-enteric adhesions. Additionally, three patients had adhesions between the epiploic appendages and the PD catheter, which led to malfunction of the PD catheter. Laparoscopic adhesiolysis was performed on all of these patients. Furthermore, laparoscopic revision was necessary due to PD catheter malfunction caused by a fibrin clot in two patients. In one case of a correct intraabdominal position of the catheter and extraperitoneal kinking, the mini laparotomy was opened additionally. In total, 15 patients after postoperative revision required a PD catheter removal due to mechanical causes during follow-up. Subsequently, a diminished median PD catheter functionality of 426 days (95% CI: 334 to 660 days) was observed in cases of postoperative revision (see Fig. S1 of the supplementary material).

In addition to postoperative revision, diabetes mellitus and female gender were identified as risk factors for a reduced technical survival of PD catheters, as indicated by elevated hazard ratios (diabetes mellitus type II: 1.44 [95% CI 0.96–2.15], female gender: 1.31 [95% CI 0.89–1.93], see Table 2). However, these results did not achieve statistical significance in the present analysis. Conversely, no association a was observed between patient age and BMI with respect to the probability of reduced technical survival of PD catheters (see Table 2). Moreover, the factors of previous abdominal surgery, prior PD catheter implantation, or intraoperative adhesiolysis did not affect the probability of reduced PD catheter survival (previous operation: 1.11; [95% CI 0.71–1.73]; *p* = 0.641, previous PD catheter implantation with 1.24; [95% CI 0.76–2.05]; *p* = 0.390, and intraoperative adhesiolysis with 0.79; [95% CI 0.30–2.10]; *p* = 0.641).

**Table 2:** Cox regression analysis for the functionality of PD catheters

Variable	Hazard ratio	95% Confidence interval	*P*-value
Age	1.01	1–1.02	0.109
Female gender	1.31	0.89–1.93	0.167
BMI	1.05	0.98–1.12	0.156
Diabetes mellitus	1.44	0.96–2.15	0.075
Adhesiolysis	0.79	0.30–2.10	0.641
Revision	**2.32**	**1.29–4.28**	**0.007**
Previous operation	1.11	0.71–1.73	0.641
Previous PD catheter	1.24	0.76–2.05	0.390

PD = peritoneal dialysis

BMI = Body mass index

### Reasons for PD catheter removal

**Fig. 3:**
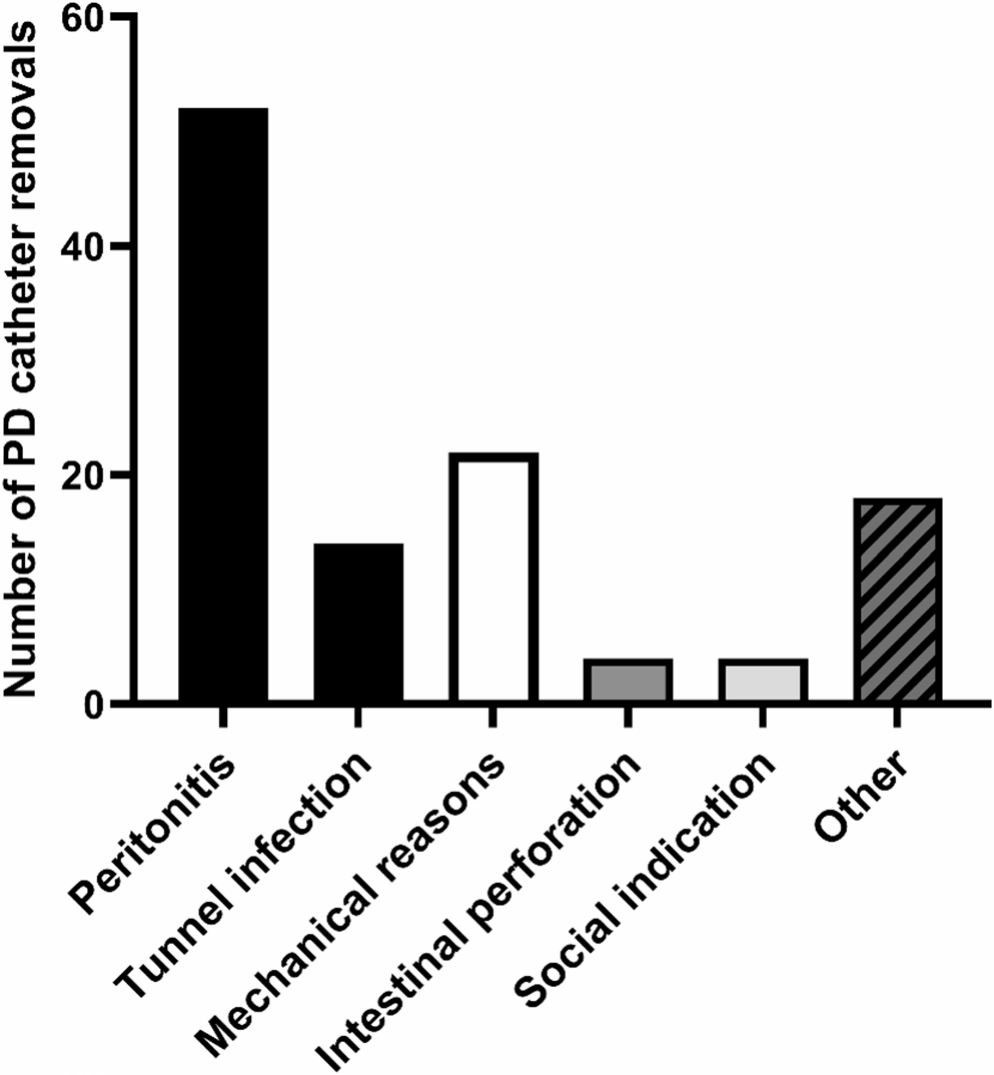
Reasons for PD catheter removal. A total of 114 PD catheter removals were necessary in 104 patients

Infectious and mechanical causes were the main reasons for PD catheter removal in our cohort. A total of 52 removals were performed due to PD catheter-associated peritonitis. Furthermore, tunnel infection led to PD catheter removal in 14 cases. In 22 cases, PD catheter removal was necessary due to mechanical causes. These causes included tip migration, adhesions, PD catheter obstruction, leakage of dialysate, pleuro-peritoneal fistula and insufficient filtration. In addition, gastrointestinal perforation associated with the PD catheter occurred in four patients. Furthermore, four PD catheters were removed due to social factors that resulted in inadequate home dialysis. The indications for PD catheter removal are illustrated in Fig. 3.

## Discussion

The present study demonstrates the functionality and technical survival of PD catheters in an extensive monocentric, retrospective cohort and identifies the underlying reasons and risk factors for their removal. Overall, a high median PD catheter functionality of 980 days [95% CI: 791 to 1095 days] was demonstrated and infectious and mechanical complications were depicted as the primary reasons for PD catheter removal. Subsequently, a postoperative revision due to a malfunction was identified as a significant risk factor for the reduced functionality of PD catheters in the three-year follow-up period, with a median PD catheter functionality of 426 days (95% CI: 334 to 660 days).

A limited number of studies have reported on the technical long-term survival rates following PD catheter revision. Compared to the findings of previous research, a study of Amerling et al. [21] showed a lower technical survival after laparoscopic revision of PD catheters with 276 days (9,2 months). In contrast, a work of Kazemzadeh et al. [22] reported a higher mean technical survival of PD catheters of 1260 days (42 months) after laparoscopic revision. Furthermore, a retrospective cohort study of Alabi et al. [23] analyzed the technical survival after laparoscopic and open revision of non-functioning PD catheters. In their study, a mean technical survival of 948 days (31,6 months) was reported after laparoscopic revision compared to 408 days (13.6months) after open revision.

In cases of intra-abdominal adhesions of the peritoneal dialysis (PD) catheter to the greater omentum, omental procedures were performed in most cases in the current study. In accordance with this, earlier studies have suggested that omental procedures, such as partial omentectomy [24, 25], omentopexy [26], or omental folding [27], may be more effective than adhesiolysis alone in ensuring the technical survival of implanted PD catheters.

In addition to the evaluation of prior abdominal surgery and intraoperative adhesiolysis, the present study is the first to examine the impact of a prior PD catheter implantation on the functionality of the subsequent implanted PD catheter. In the current study cohort, 12% of the patients had previously undergone PD catheter implantation. However, the present study found that previous PD catheter implantation was not a significant risk factor for reduced functionality of the subsequent PD catheter. Moreover, the BMI of patients was not associated with a reduced technical survival, as already indicated by previous research [18]. Furthermore, the results of the present study support the findings that prior surgical procedures [16, 17] and the necessity for intraoperative adhesiolysis are not associated with increased rates of PD catheter removal and reduced functionality. Overall, only 4% of patients required laparoscopic adhesiolysis prior to PD catheter implantation. In previous studies on laparoscopic PD catheter implantation, it was reported that laparoscopic adhesiolysis was required prior to PD catheter implantation in 26.9–31.8% of cases [16, 17]. Consequently, an advantage of the surgical technique described in this study can be discussed, as the need for adhesiolysis was reduced compared to laparoscopic PD catheter implantation.

Despite the significant findings mentioned previously, this study has some limitations. First, it is highly probable that postoperative revision is solely a confounder for the underlying cause of PD catheter dysfunction. Consequently, a postoperative revision may suggest a mechanical dysfunction of a PD catheter, thereby serving as a rescue approach prior to the removal of the PD catheter. Furthermore, due to the retrospective study design, several patient characteristics such as socioeconomic status and education level were not considered, although they have been discussed in previous studies as possible risk factors for PD catheter-associated peritonitis and thus reduced PD catheter functionality [28–30]. In addition, a lower socioeconomic status and education level may increase the risk of inadequate home dialysis and thus a social indication for PD catheter removal. Accordingly, a social indication for PD catheter removal was identified in four patients within our retrospective cohort.

Several studies have previously identified diabetes mellitus as a risk factor for PD catheter-associated peritonitis and tunnel infection, often leading to the removal of the PD catheter [5, 9]. While the present study determined a higher hazard ratio for diabetes mellitus as a risk factor for PD catheter removal, these findings were not statistically significant.

Moreover, a high number of participants withdrew during the follow-up period due to the retrospective study design. In addition, a high mortality rate was observed during the observation period, with 59 deaths, corresponding to 17% of the total study cohort. Two deaths were attributable to a perforation of the gastrointestinal tract associated with a PD catheter. Consequently, in this retrospective study, there is a strong suspicion of selection bias, since particularly ill patients were observed in the long-term follow-up. However, a median PD catheter functionality of 980 days was calculated in the present cohort, which corresponds to a notably prolonged lifespan of PD catheters, particularly in this multimorbid patient cohort [31].

In conclusion, peritoneal dialysis can be considered a safe treatment option for patients with end-stage renal disease, and the implanted PD catheters demonstrated long-term functionality in the present study. As demonstrated in earlier research, peritoneal dialysis has been shown to have several advantages over hemodialysis. For instance, patients undergoing continuous ambulatory peritoneal dialysis (CAPD) or continuous cycling peritoneal dialysis (CCPD) reported a higher quality of life compared to those undergoing thrice-weekly hemodialysis. The most important reason for the improved quality of life is greater patient autonomy [32–34]. Moreover, peritoneal dialysis proved more cost-effective compared to hemodialysis in previous studies. In high-income countries, the financial burden of frequent hemodialysis is reported to be 1.25 to 2.35 times greater than that of peritoneal dialysis, primarily due to the requirement of assistance from dialysis staff and the high transportation costs to dialysis centers [35]. Consequently, peritoneal dialysis is a recommended treatment for patients with end-stage renal disease as “PD first” concept.

## Conclusion

In summary, successful PD catheter implantation was achieved in 340 patients, with a median PD catheter functionality of 980 days, and only 4% of the retrospective study cohort requiring laparoscopic adhesiolysis prior to PD catheter implantation. Postoperative revision due to mechanical reasons was the only risk factor that was significantly associated with PD catheter removal. Consequently, peritoneal dialysis can be safely offered for patients with end-stage renal disease, regardless of previous PD catheter implantation, intraoperative adhesiolysis, or previous abdominal surgery.

## Supplementary Information

Below is the link to the electronic supplementary material.

Supplementary Material 1

Supplementary figure 1 Fig. S1 Kaplan-Meier curve illustrating the functionality of PD catheters with postoperative revision over time after implantation. The ticks represent loss-of-follow-up. (PNG 50.0 kb)

High Resolution Image (TIF 321 kb)

## Data Availability

Collected data are available from the corresponding author on request.
