# Supplementary material for: Surgical risk factors for technical survival of peritoneal dialysis catheters
Source: Langenbecks Arch Surg. 2025 Nov 10;411(1):8. doi: 10.1007/s00423-025-03901-7 (PMC12602561; doi:10.1007/s00423-025-03901-7)
Supplement: Supplementary file 1 — Supplementary Material 1 [file 423_2025_3901_MOESM1_ESM.docx]

| Year | Number of Patients | Number of PD catheter implantations |
| --- | --- | --- |
| 2010 (Begin: January 2010) | 23 | 23 |
| 2011 | 21 | 21 |
| 2012 | 27 | 27 |
| 2013 | 29 | 29 |
| 2014 | 25 | 27 |
| 2015 | 38 | 41 |
| 2016 | 36 | 40 |
| 2017 | 28 | 31 |
| 2018 | 23 | 24 |
| 2019 | 20 | 21 |
| 2020 | 35 | 40 |
| 2021 | 27 | 28 |
| 2022 (End: March 2022) | 8 | 8 |
| Overall | 340 | 360 |
