# Supplementary figures and images for: Surgical risk factors for technical survival of peritoneal dialysis catheters
Source: Langenbecks Arch Surg. 2025 Nov 10;411(1):8. doi: 10.1007/s00423-025-03901-7 (PMC12602561; doi:10.1007/s00423-025-03901-7)

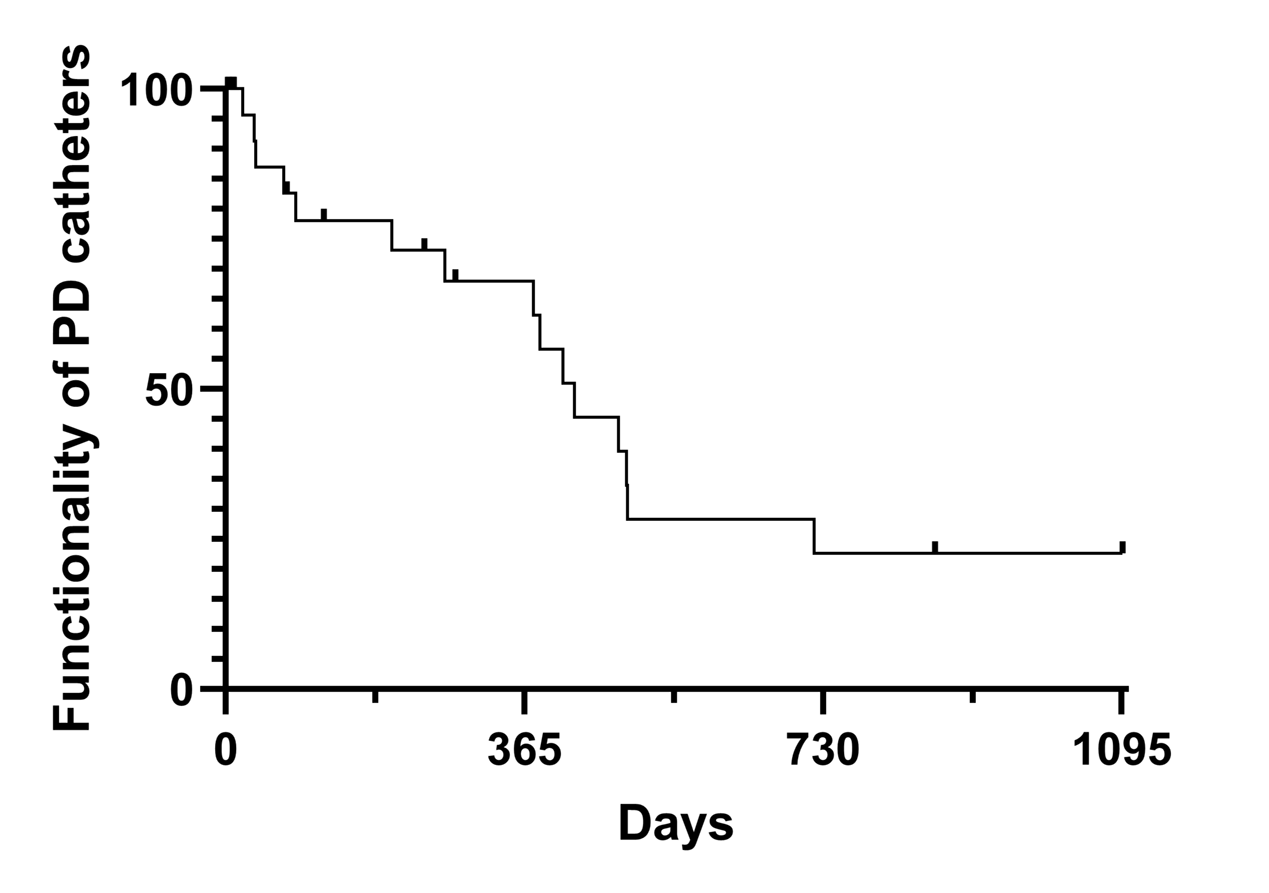

Supplement: Supplementary file 2 — Fig. S1 Kaplan-Meier curve illustrating the functionality of PD catheters with postoperative revision over time after implantation. The ticks represent loss-of-follow-up. (PNG 50.0 kb) [file 423_2025_3901_Fig4_ESM.png]

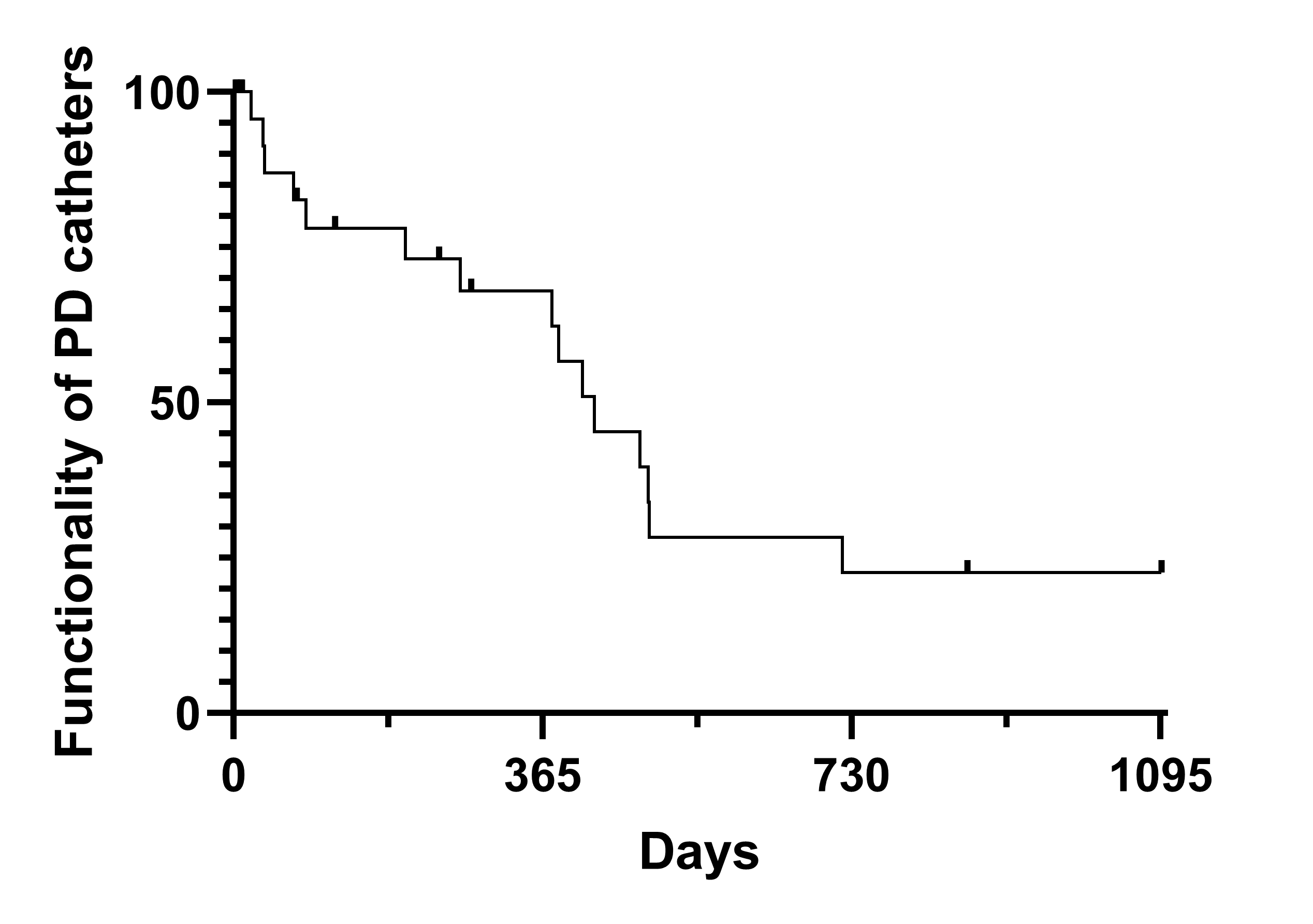

Supplement: Supplementary file 3 — High Resolution Image (TIF 321 kb) [file 423_2025_3901_MOESM2_ESM.tif]
